# Success of Current COVID-19 Vaccine Strategies vs. the Epitope Topology of SARS-CoV-2 Spike Protein-Receptor Binding Domain (RBD): A Computational Study of RBD Topology to Guide Future Vaccine Design

**DOI:** 10.3390/vaccines10060841

**Published:** 2022-05-25

**Authors:** Santhinissi Addala, Madhuri Vissapragada, Madhumita Aggunna, Niharikha Mukala, Manisha Lanka, Shyamkumar Gampa, Manikanta Sodasani, Jahnavi Chintalapati, Akhila Kamidi, Ravindra P. Veeranna, Ravikiran S. Yedidi

**Affiliations:** 1 Stem Cell Biology Branch, Department of Intramural Research Core, The Center for Advanced-Applied Biological Sciences & Entrepreneurship (TCABS-E), Visakhapatnam 530016, India; santhinissiaddala@gmail.com; 2 Synthetic Biology Branch, Department of Intramural Research Core, The Center for Advanced-Applied Biological Sciences & Entrepreneurship (TCABS-E), Visakhapatnam 530016, India; vissapragadamadhuri5@gmail.com; 3 Multiomics-Oncology & Immunotherapy Branch, Department of Intramural Research Core, The Center for Advanced-Applied Biological Sciences & Entrepreneurship (TCABS-E), Visakhapatnam 530016, India; aggunnamadhumita@gmail.com; 4 Multiomics-Infectious Diseases Branch, Department of Intramural Research Core, The Center for Advanced-Applied Biological Sciences & Entrepreneurship (TCABS-E), Visakhapatnam 530016, India; jessicaneha20@gmail.com; 5 Department of Intramural Research Core, The Center for Advanced-Applied Biological Sciences & Entrepreneurship (TCABS-E), Visakhapatnam 530016, India; manishalanka98@gmail.com (M.L.); gngshyam@gmail.com (S.G.); mani81314821@gmail.com (M.S.); janu2001bg@gmail.com (J.C.); akhilakamidi05@gmail.com (A.K.); 6 Department of Biochemistry, Central Food Technological Research Institute, Mysore 570020, India; 7 Department of Zoology, Andhra University, Visakhapatnam 530003, India

**Keywords:** COVID-19, SARS CoV-2, vaccines, spike protein, ACE-2 receptor, epitope

## Abstract

Coronavirus disease-2019 (COVID-19) is a pandemic with a high morbidity rate occurring over recent years. COVID-19 is caused by the severe acute respiratory syndrome causing coronavirus type-2 (SARS-CoV-2). COVID-19 not only challenged mankind but also gave scope to the evolution of various vaccine design technologies. Although these vaccines protected and saved many lives, with the emerging viral strains, some of the strains may pose a threat to the currently existing vaccine design that is primarily based on the wild type spike protein of SARS-CoV-2. To evaluate the risk involved from such mutant viral strains, we performed a systematic in silico amino acid substitution of critical residues in the receptor binding domain (RBD) of the spike protein. Our molecular modeling analysis revealed significant topological changes in the RBD of spike protein suggesting that they could potentially contribute to the loss of antigen specificity for the currently existing therapeutic antibodies/vaccines, thus posing a challenge to the current vaccine strategies that are based on wild type viral spike protein epitopes. The structural deviations discussed in this article should be considered carefully in the future vaccine design.

## 1. Introduction

**Figure 1:**
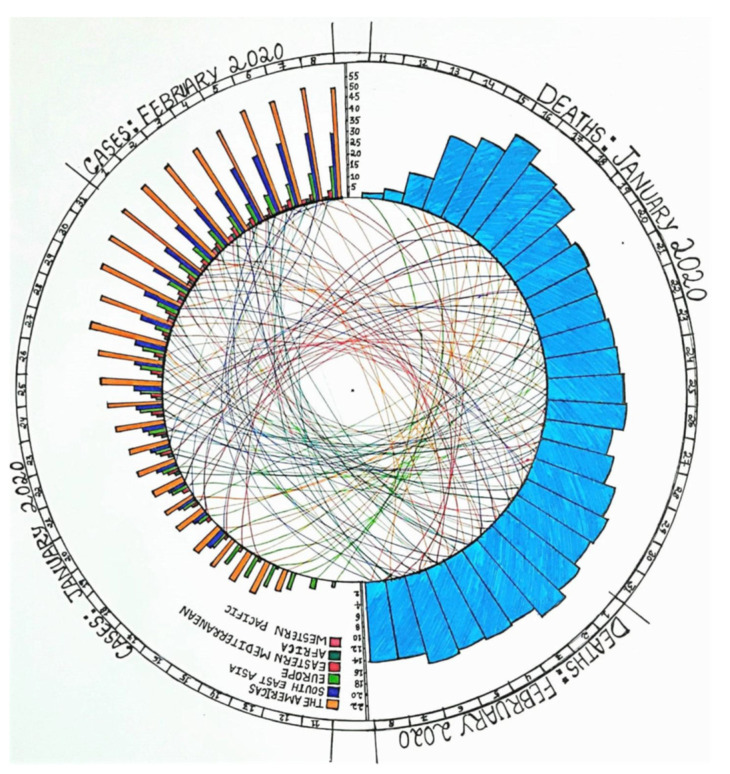
**Initial epidemiology of COVID-19 in 2020.** This circular plot represents the progressive increase in the number of infections/cases in the beginning of 2020 world wide. The left half of the circle shows the number of increasing cases per country over a period of one month (11 January to 8 February 2020). Right half of the circle shows the number of deaths over the same period of time from the countries listed taken together. Correlations are shown as lines in the center of the circle connecting the relevant data points from two halves of the circle.

Coronavirus disease-2019 (COVID-19), a contagious viral disease that affects the respiratory tract, is caused by Severe Acute Respiratory Syndrome Coronavirus type-2 (SARS CoV-2) [1]. Since its first identification in Wuhan, China 2019, it has posed a health emergency causing high morbidity across the world (Figure 1) [2]. Considering the effects of the SARS CoV-2 outbreak on the livelihood of people and on the global economy, the World Health Organization (WHO) declared it to be a pandemic on 11 March 2020 [3]. SARS CoV-2 is an RNA virus and belongs to the genus Betacoronavirus which includes ++ssRNA, and enveloped viruses such as Middle east respiratory syndrome (MERS) [4,5]. Structurally SARS CoV-2 has Spike (S), Envelope (E), Membrane (M) which together form a viral envelope and N (nucleocapsid) protein that holds RNA [6]. S protein is 1273 aa long and is coded by the nucleotides between 21,563 and 25,384 from the whole genome of around 30,000 bp [7,8]. Spike protein consists of S1 and S2 subunits which perform different functions yet play a role together in viral entry into the host cell [9]. S1 subunit region is responsible for interacting with the human Angiotensin Converting enzyme-2 receptor (hACE-2r) through the Receptor Binding Domain (RBD), whereas S2 is a well conserved region with fusion peptides that fuse viral and host cell membranes, thus facilitating viral entry [10]. Apart from the S protein, SARS CoV-2 also contains furin cleavage at the junction of S1 and S2 which is absent in SARS CoV-1 [11]. The crown-like appearance of this virus is due to the spike protein the present outside of the viral particle, thus making it a potential target for several vaccines that are developed [7,12,13] and the production of neutralizing antibodies [14,15] towards plasma therapy (for passive immunity), which initially is recommended for treatment [16]. However, considering other aspects, plasma therapy is only recommended in extreme conditions [16,17,18]. Vaccines, on the other hand, contribute to active immunity developed until now and mostly target the epitope of the spike protein [19,20]. 

Traditionally, vaccines have been prepared by heat-killing or weakening (called attenuation) of the disease causing microbe. However, agents that resemble the disease causing organism, toxin, or its surface protein have also been used via recombinant DNA technology [21]. Despite several misinterpretations of the general public, vaccines are considered to be one of the best ways to prevent an infection [22] administered either as a prophylactic or a therapeutic measure to prevent or fight disease [21,22,23]. Inactivated vaccines are prepared using the killed form of the disease causing organisms (virus particles, bacterial pathogens, etc.) which are culture grown to destroy the disease producing capacity of the agent but can elicit an immune response for a specific disease [23,24,25]. Comparatively, the immune response produced by an inactivated vaccine is weaker and it may need more than one dose or multiple booster doses for an effective immune response [24,25,26,27]. In the case of COVID-19, caused by SARS-CoV-2, COVAXIN is an inactivated vaccine developed using whole-virion inactivated vero cell derived platform technology [28,29,30]. Live-attenuated vaccines can elicit stronger immune responses against the pathogen and require multiple or a single dose of vaccination to provide lifelong immunity [31]. 

The mRNA vaccine does not contain the disease causing organism, hence the person receiving an mRNA vaccine neither becomes exposed to the disease -ausing agent through the vaccine, nor can they become infected because of the vaccine components, unlike inactivated or live vaccines, which can have a chance of containing live disease-causing agents and can pose a threat of infection if not prepared properly [32,33]. In the context of SARS-CoV-2, the vaccines from Pfizer-Biotech and Moderna are mRNA vaccines and are also the first COVID-19 vaccines which achieved authorization and approval for their usage in the United States with >90% efficacy in clinical trials [34,35,36]. Vaccine types such as—subunit, recombinant, polysaccharide, and conjugate vaccines are used to protect against diseases such as Haemophilus influenzae type b, Hepatitis B, HPV, Pneumococcal disease etc. [37,38,39,40,41]. In the case of diseases such as diphtheria, tetanus etc., vaccines are prepared based on the toxins produced by the organism [42]. Toxoid vaccines elicit an immune response against the toxins which are responsible for the disease [42,43]. Adenovirus has been used as the vector for the development of the vaccine against COVID-19 by Oxford-AstraZeneca [44,45,46,47].

The Receptor Binding Domain (RBD) from the S1 subunit of spike protein interacts directly with the hACE-2r. X-ray crystallographic analysis revealed that residues Arg319-Phe541 contribute to the RBD of SARS CoV-2, which has some similar features to the RBD of SARS CoV-1 [48]. It is identified that 13 hydrogen bonds and two salt bridges are formed at the interface between RBD-hACE-2r [48]. Vaccines that are developed based on RBD are also found to be highly immunogenic and can thus prevent viral entry and fusion with host cells [49]. This explains the robustness of RBD in causing COVID-19. However, mutations in the spike protein may lead to changes in structural conformation that can affect the affinity of RBD-hACE-2r which in turn leads to change in viral fitness (defined as viral replication and infectivity of a mutant strain either in par with or better than the wild type). This may lead to a failure of vaccines that are specifically developed against a specific antigenic determinant. In this study, eleven amino acid positions (Lys417, Gly446, Tyr449, Asn487, Gln493, Gly496, Gln498, Thr500, Asn501, Gly502, and Tyr505) that are located on the RBD of the SARS CoV-2 spike protein that make direct contacts with the hACE-2r were targeted for systematic in silico mutational analysis at mRNA level as well as the protein level. At each position all possible combinations of the triplet codons were evaluated one at a time to obtain the mRNA stability using the RNAfold web server followed by all possible amino acid substitutions at each position to understand the structural and topological changes in the RBD of the viral spike protein. Together with the mRNA mutants’ stability in combination with the protein structural deviations, we calculated the viral fitness that is indicative of high survival rates compared to the wild type strain.

## 2. Materials and Methods

### 2.1. Preparation of Mutant RNA and Protein Sequences

**Figure 2:**
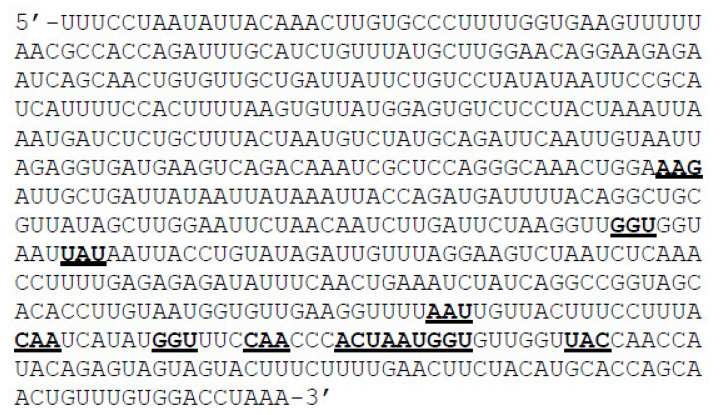
**Viral mRNA sequence that was considered in this study.** In this sequence, the starting triplet codon UUU was taken as the first codon and hence the corresponding amino acid for UUU was taken as the first amino acid in this study. Triplets that were analyzed in this study are highlighted in bold font and were underlined.

The RBD nucleotide sequence of wild type SARS-CoV-2 spike protein (Figure 2) was taken as the working template in which the triplet codons for the proposed 11 positions were systematically mutated one base at a time. For example, if the wild type codon sequence is AAG then the systematic point mutations would generate nine possible combinations: AAA, AAC, AAU, ACG, AUG, AGG, CAG, GAG, and UAG. Each mutant sequence was saved as a separate file that can be copy-pasted into the online RNAfold web server (http://rna.tbi.univie.ac.at/). Once the mutant RNA sequences were generated, their corresponding amino acid sequences were also manually generated separately using the standard codon dictionary. Separate files for mutant amino acid sequences are convenient for copy-pasting them into the SWISS MODEL server (https://swissmodel.expasy.org) in order to generate the mutant protein models.

### 2.2. RNA Folding and Energy Calculations

Each mutant sequence was copy pasted into the RNAfold web server (http://rna.tbi.univie.ac.at/; accessed on 1 January 2022) in order to calculate the mRNA secondary structure. The secondary structure of the mRNA mutant molecule was downloaded along with the minimum free energy (MFE) values and ensemble diversity (ED) numbers. After analyzing the MFE values it was found that there were variations in the MFE values as well as the ED numbers. To maintain uniformity, the MFE value of each mutant secondary structure was divided by its corresponding ED number to obtain the average MFE (AMFE) values per iteration of the secondary structure ensemble. These AMFE values of each mutant were then compared to that of the wild type to calculate the viral fitness, taking the wild type AMFE as 100%.

### 2.3. Generation of Mutant Protein Models

The mutant protein amino acid sequences as prepared above were submitted to the online SWISS MODEL server (https://swissmodel.expasy.org/, accessed on 1 January 2022) in order to build the 3D models. For model building, an analysis was made as described previously [50]. Briefly, each mutant model was superposed onto the wild type model using the CCP4 software to evaluate the backbone structural deviations taking the wild type Cα positions as reference. The root mean square deviations (RMSDs) (*y*-axis) were then plotted vs. the amino acid positions taken on the *x*-axis.

### 2.4. Structural Analysis

Hydrogen bond pattern analysis between the human ACE-2 receptor (hACE-2r) and each of the mutant models of the viral receptor binding domain (RBD) was performed using PyMOL molecular graphics software. Amino acid substitutions in the various mutant models showed either loss/gain of hydrogen bonds or no change in the profile as compared to the wild type model that was used as a reference. 

### 2.5. Binding Affinity

Binding affinity values were calculated based on the number of hydrogen bonds between the hACE-2r and RBD for each of the mutant models in this study. This protocol considers the enthalpic contributions of the hydrogen bonds towards the binding affinity thus ignoring the entropic contributions. However, the binding affinity values calculated here are only relative to the wild type RBD-hACE-2r interface with only one amino acid substitution which may not generate significant differences in the relative entropic components of the mutants when compared to the wild type. Furthermore, the molecular surface area calculations for each of the mutant models including the wild type were performed using CCP4 software.

### 2.6. Viral Fitness

Viral fitness in general is defined as the collective viral replication capacity and viral infectivity rates both of which are in vitro and/or in vivo studies. In this in silico study, we considered three factors, such as mRNA stability, number of hydrogen bonds at the RBD-hACE-2r interface and structural deviations in the mutant RBD models generated by the amino acid substitutions as contributors to the successful viral replication and infectivity. Evidently, the wild type virus already created a pandemic thus proving that it has 100% viral fitness. By taking the three factors from wild type, we calculated the relative viral fitness of the mutants that were analyzed in this study.

## 3. Results and Discussion

### 3.1. Mutant mRNA Sequences Show Higher Stability than Wild Type

The full length of 600 base pairs RBD mRNA nucleotide sequence that was considered for this study (Figure 2) was divided into 200 triplet codons for easy analysis by starting from the first base at the 5’-end of the sequence (Figure 2). As shown in Table S1, the eleven triplet codons at positions 89, 118, 121, 159, 165, 168, 170, 172, 173, 174, and 177 that are critical in the RBD were chosen for mutagenesis. At each of the eleven positions selected, 6 to 8 mutant triplet codons were substituted one at a time to evaluate the mRNA stability. As shown in Table S1, the overall minimum free energy (MFE) of the mutant mRNA sequences ranged from −179.07 kcal/mol to −189.25 kcal/mol. compared to the wild type RBD mRNA sequence (−183.78 kcal/mol). Since the MFE values are reflective of their respective thermodynamic ensembles and each mutant has different ensemble diversities ranging from 75.92 to 131.06, we took the average MFE (AMFE) values for better representation of the MFE values per each mutant mRNA in comparison with the wild type mRNA. 

**Figure 3:**
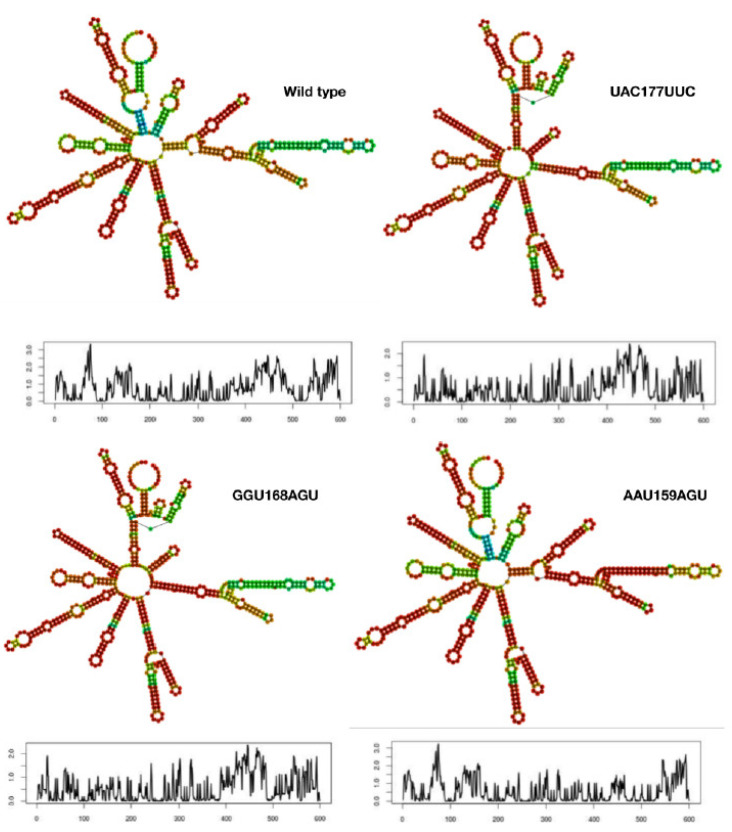
**Secondary structure and stability of mRNA.** The secondary structure of three mutants are compared to the wild type along with their entropic profiles obtained from the RNAfold server. The entropic profiles were plotted with position of the base taken on *x*-axis vs. entropy on the *y*-axis. Evidently all four show different profiles for their secondary structures as well as their entropic distributions.

Comparative analysis of the AMFE values revealed three mutant triplets (AAU159AGU, GGU168AGU, and UAC177UUC) in particular that have higher values compared to the wild type (Table S1). The secondary structure of mRNA for these three mutants and the wild type are shown in Figure 3 along with their corresponding entropic profiles. The mutant AAU159AGU resembled the wild type mRNA while the other mutants showed more similarities with each other rather than with the wild type mRNA. The mutants GGU168AGU and UAC177UUC not only showed similarities in their mRNA secondary structures with each other but also exhibited similar entropic profiles (Figure 3). The entropic profile of AAU159AGU mutant showed some similarity to that of the wild type but is evidently different from the other two mutants.

Typically, the stability of mRNA in the host cytoplasm is directly proportional to its secondary structure; in other words, the AMFE value among several other factors. The AMFE values are sequence dependent and hence they differ for wild type and mutants, suggesting the relative stability of the corresponding mRNA. Additionally, the longevity of mRNA in the host cytoplasm depends on its stability. The longer the mRNA exists in the host cytoplasm, the higher the chances of more rounds of the spike protein translation which in turn helps package and release of the new virions (increased viral fitness compared to the wild type). Hence, the mRNA stability was considered to be one of the important factors in this study. 

**Figure 4:**
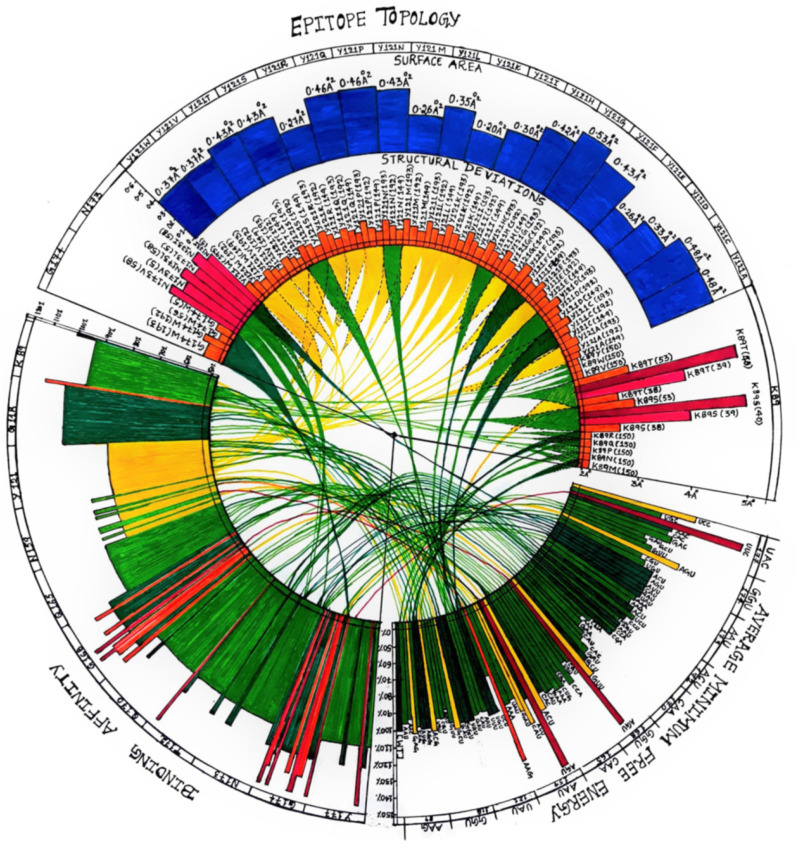

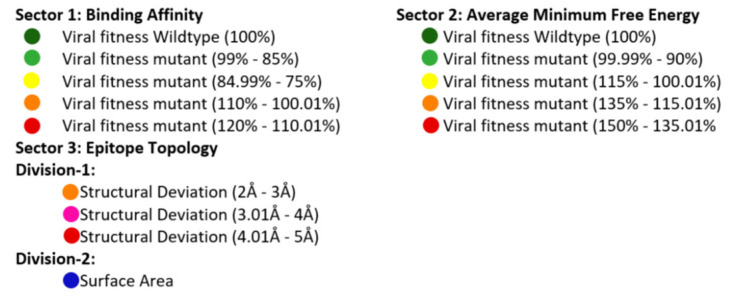
**Circular plot correlating mRNA vs. protein parameters.** The circular plot shows correlation between the mRNA stability and AMFE-based viral fitness (**bottom right sector**) vs. binding affinity-based viral fitness (**bottom left sector**) vs. structural deviations and changes in the epitope topology (**top sector**).

Furthermore, the AMFE values of all the mutants (Table S1) were used to arbitrarily predict the relative viral fitness of those mutants by taking the wild type as 100%. As shown in Table S1, the above-mentioned three mutants were found to have higher relative viral fitness roughly 1.4-fold higher than the wild type viral fitness. This increase in the viral fitness may not seem to be dangerous, but when these calculations are compared with the structural deviations of various mutant RBDs, then a comprehensive conclusion can be made with respect to the threat levels posed by these mutants against the current vaccines that are based on wild type RBD. The viral fitness values of all the chosen mutant models that were calculated based on the AMFE values are conveniently plotted in a circular plot as shown in Figure 4. The mutants exhibiting higher relative viral fitness values are represented by red bars followed by orange and yellow bars while the mutants that exhibited relative viral fitness similar to the wild type are shown as dark green bars. Mutants that exhibited lower relative viral fitness values compared to the wild type are indicated by the light green bars. In summary, out of the 74 mutants represented in the circular plot (Figure 4), around 33 mutants (~45%) exhibited lower relative viral fitness compared to the wild type while the rest exhibited either equivalent or higher relative viral fitness compared to the wild type.

### 3.2. Mutants Show Altered RBD-hACE-2r Interface Hydrogen Bonds

**Figure 5:**
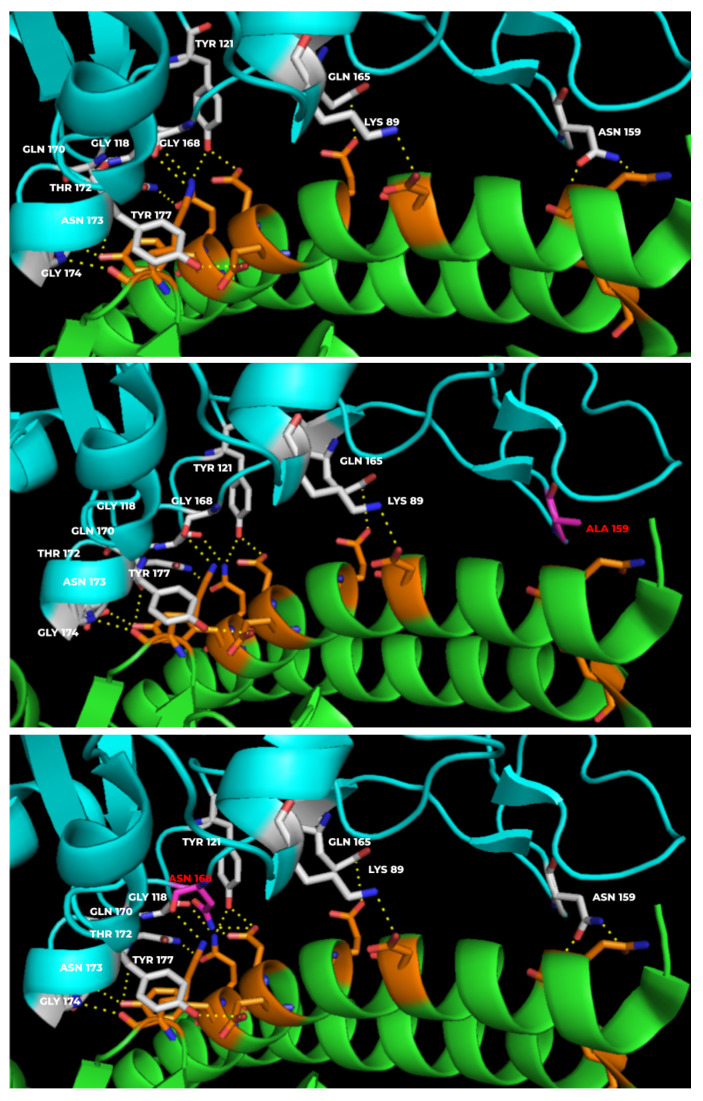
**Hydrogen bonding pattern at the RBD-hACE-2r interface.** The top panel shows a wild type interface with 13 hydrogen bonds while the middle and bottom panels show the interfaces of N159A (11 hydrogen bonds) and G168N (15 hydrogen bonds), respectively. The two mutant interfaces represent loss and gain of hydrogen bonds in the middle and bottom panels, respectively.

The mutant RBD-hACE-2r interface hydrogen bond profiles were analyzed to understand any changes in the hydrogen bonding pattern of the mutants compared to the wild type. As shown in Figure 5, the wild type showed 13 hydrogen bonds at the interface while the mutants N159A and G168N showed 11 and 15 hydrogen bonds, respectively, at the interface. The hydrogen bonding patterns for mutants were analyzed by systematically substituting all the eleven positions (Lys417, Gly446, Tyr449, Asn487, Gln493, Gly496, Gln498, Thr500, Asn501, Gly502, Tyr505) with all the 20 natural amino acids one amino acid at a time (Table S2). The amino acid numbering given in Table S2 is according to the numbering system followed in Figure 2. Interestingly, only selected amino acid substitutions showed changes in the hydrogen bonding pattern, so instead of analyzing the hydrogen bonding patterns for all the mutants, we calculated the binding affinity-based predicted relative viral fitness given in Table S2 and Figure 4. 

The wild type with 13 hydrogen bonds at the interface was taken as 100% fit; accordingly, any increase or decrease in hydrogen bonds at the interface for the mutants was calculated as relatively higher fitness or lower fitness compared to the wild type. Lys417 (K89 in this study) showed an increase in viral fitness only for the K89Y substitution. Gly446 (G118 in this study) showed equal viral fitness with any amino acid substitution at that position. Tyr449 (Y121 in this study), Asn487 (N159 in this study) and Gln493 (Q165 in this study) showed decreased viral fitness with any of the 20 amino acid substitutions. Gly496 (G168 in this study) showed either equal or higher viral fitness compared to the wild type. Especially G168N and G168R showed the highest viral fitness among the 20 amino acid substitutions at that position. While Gln498 (Q170 in this study) and Asn501 (N173 in this study) showed either equal or lesser viral fitness compared to the wild type, Gly502 (G174 in this study) showed either equal or higher viral fitness in comparison to wild type. In particular, the substitutions G174D/H/Q/R/Y showed the highest viral fitness (115.4%) and G174E/N/S/T showed higher viral fitness (107.7%) than wild type but relatively lower fitness than the aforementioned substitutions. In the case of Thr500 (T172 in this study) and Tyr505 (Y177 in this study) all the substitutions showed either equal or lesser viral fitness compared to the wild type. However, the amino acid substitutions T172D and Y177R both showed highest viral fitness values (115.4%) compared to the wild type. Overall, 9 substitutions showed highest viral fitness (115.4%) compared to wild type (Table S2). As shown in Figure 4, the predicted viral fitness values from the interface hydrogen bonding patterns can be easily compared and contrasted all together in one circular plot. Even though the viral fitness values are arbitrary, the relative values compared with various mutant models gave us a comprehensive outlook of how these amino acid substitutions play a role in the binding affinity of the viral spike protein to the host ACE-2 receptor. We further investigated any possible structural deviations in the RBD that might be caused by the amino acid substitutions.

### 3.3. Mutant Models Show Structural Deviations Onsite and Offsite

**Figure 6:**
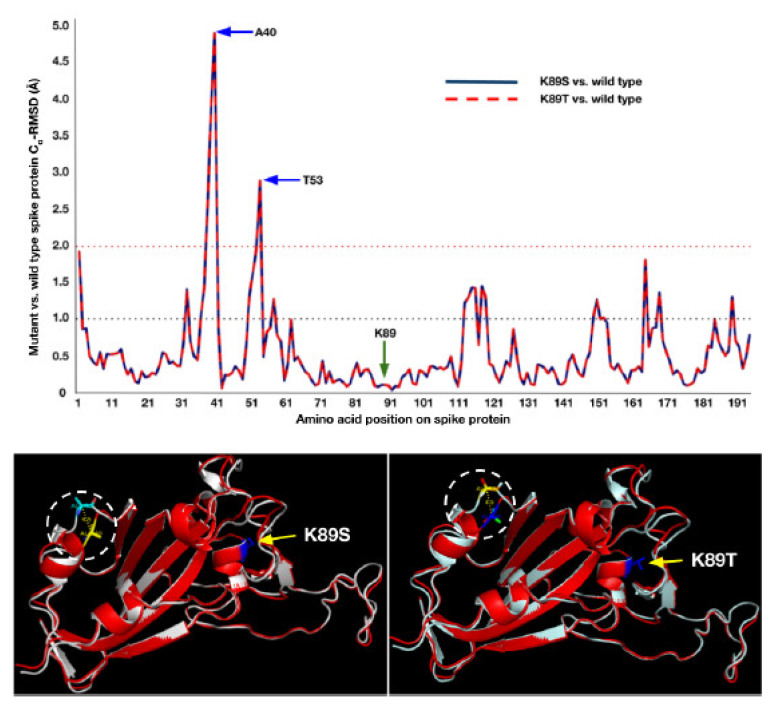
**Structural deviation in the RBD of K89S/T mutants.** The top panel shows RMSD of Cα atoms of mutants superposed onto wild type. Significant deviations were seen at positions 40 and 53. Bottom panels show the offsite structural deviations at position 40 on the respective mutant RBDs highlighted by white dashed circles.

In addition to the mRNA stability analysis, we further complimented the study with the three-dimensional structural deviations in the mutant models compared to the wild type model. As shown in Table S3, most of the amino acid substitutions did not yield the Cα RMSDs beyond 2 Å especially at the site of amino acid substitution. Interestingly, however, there were offsite RMSDs observed for certain mutants such as K89S/T and N173V/I/L that showed significant RMSDs ranging beyond 3 Å (Figure 4). As shown in Figure 6, the K89S/T mutants did not show any RMSD at the site of amino acid substitution but instead exhibited two major RMSDs at positions 40 (>4.5 Å) and 53 (3 Å). These major structural deviations cause significant epitope topology changes due to which the therapeutic antibody binding may be affected severely. We believe that it is safe to assume that the antibodies generated in the vaccinated individuals can typically encounter and neutralize the wild type viral spike protein that may have structural deviations <2 Å (Table S3) as these deviations fall within the range of protein flexibility, Luzzati error, etc. However, mutants such as K89S/T pose a serious threat to the currently existing vaccine generated antibodies. Our current study does not take into consideration the flexibility of the mutant proteins which will be analyzed in future using molecular dynamics simulations both in the presence and absence of the hACE-2r in order to obtain a complete understanding of the real binding affinities of the mutants for correlating them with their viral fitness. The current study is completely a computational modeling-based predictive study and the conclusions in this study are to be tested and evaluated in future with appropriate supporting experiments such as in vitro binding affinities of mutant RBDs against the hACE-2r using various Biochemical/Biophysical techniques.

## Data Availability

Not applicable.

## Supplementary Materials

The following supporting information can be downloaded at: https://www.mdpi.com/article/10.3390/vaccines10060841/s1, Table S1: Systematic base-substitutions in RBD mRNA sequence and RNAfold parameters; Table S2: Viral fitness (%) for mutants based on the binding affinity calculations from the number of hydrogen bonds at the RBD-ACE2 receptor binding interface compared to the wild type (taken as 100%); Table S3: Structural deviations that are >2.0 Å and <3.0 Å seen in mutants based on the C_α_ RMS deviations compared to the wild type at the site of mutation vs. off-sites.
