# Supplementary material for: Success of Current COVID-19 Vaccine Strategies vs. the Epitope Topology of SARS-CoV-2 Spike Protein-Receptor Binding Domain (RBD): A Computational Study of RBD Topology to Guide Future Vaccine Design
Source: Vaccines (Basel). 2022 May 25;10(6):841. doi: 10.3390/vaccines10060841 (PMC9228374; doi:10.3390/vaccines10060841)
Supplement: Supplementary file 1 [file vaccines-10-00841-s001.zip › vaccines-1682785-supplementary.pdf]

SUPPLEMENTARY DATA

Success of current COVID-19 vaccine strategies vs. the epitope topology of SARS-CoV-2 spike protein-receptor binding domain (RBD): A computational study of RBD topology to guide future vaccine design.

Santhinissi Addala[1, #], Madhuri Vissapragada[2, #], Madhumita Aggunna[3], Niharikha Mukala[4], Manisha Lanka[5], Shyamkumar Gampa[5], Manikanta Sodasani[5], Jahnavi Chintalapati[5], Akhila Kamidi[5], Ravindra P. Veeranna[6, *] and Ravikiran S. Yedidi[1, 2, 3, 4, 5, 7] *

Affiliations:

[1]Stem Cell Biology branch, [2]Synthetic Biology branch, [3]Multiomics-Oncology & Immunotherapy branch, [4]Multiomics-Infectious Diseases branch, [5]Department of Intramural research core, The Center for Advanced-Applied Biological Sciences & Entrepreneurship (TCABS-E), Visakhapatnam 530016, A.P. India; [6]Department of Biochemistry, Central Food Technological Research Institute, Mysore 570020, K.A. India. [6]Department of Zoology, Andhra University, Visakhapatnam 530003, A.P. India. [#]Contributed equally.

*Correspondence: R.S.Y.: tcabse.india@gmail.com & R.P.V.: raviravindra1@gmail.com

Postal address:

Ravikiran S. Yedidi, Ph.D.

Department of Intramural Research Core
The Center for Advanced-Applied Biological Sciences & Entrepreneurship (TCABS-E)
Door No. 47-11-19/19, Fourth floor
Seethammapeta Main Road, Dwarakanagar
Visakhapatnam. 530016
Phone: +91-8660301662
E-mail: tcabse.india@gmail.com

SUPPLEMENTARY DATA

Table S1. Systematic base-substitutions in RBD mRNA sequence and RNAfold parameters.

Position	Wildtype triplet	Mutant	Minimum free energy (kcal/mol)	Ensemble diversity	*AMFE (kcal/mol)	#Viral fitness (%)
89	AAG	ACG	-185.89	108.32	-1.71	101.18
		AGG	-184.44	108.74	-1.69	100.00
		AUG	-184.22	108.4	-1.69	100.00
		CAG	-183.3	108.72	-1.68	99.41
		GAG	-186.89	108.31	-1.72	101.77
		AAC	-180.14	112.93	-1.59	94.08
		AAU	-180.16	109.24	-1.64	97.04
118	GGU	GGU	-183.78	108.3	-1.69	100.00
		GAU	-183.29	108.32	-1.69	100.00
		GCU	-186.22	107.57	-1.73	102.37
		GUU	-185.21	110.05	-1.68	99.41
		AGU	-181.38	118.36	-1.53	90.53
		CGU	-181.12	113.11	-1.6	94.67
		UGU	-181.35	114.16	-1.58	93.50
121	UAU	UCU	-182.02	134.75	-1.35	79.88
		UGU	-183.81	108.42	-1.69	100.00
		UUU	-182.7	107.92	-1.69	100.00
		AAU	-181.19	110.01	-1.64	97.04
		CAU	-181.17	109.54	-1.65	97.63
		GAU	-182.2	108.86	-1.67	98.81
159	AAU	ACU	-184.24	98.09	-1.87	110.65
		AGU	-189.25	77.2	**-2.45**	144.97
		AUU	-183.77	107.63	-1.7	100.59
		CAU	-184.21	94.86	-1.94	114.79
		GAU	-186.13	104.52	-1.78	105.32
		UAU	-183.55	112.65	-1.62	95.85
		AAA	-183.54	110.71	-1.65	97.63
		AAG	-185.49	82.61	-2.24	132.54
165	CAA	CCA	-184.14	102.59	-1.79	105.91
		CGA	-183.56	114	-1.61	95.26

SUPPLEMENTARY DATA

		CUA	-182.98	103.85	-1.76	104.14
		AAA	-183.1	103.19	-1.77	104.73
		GAA	-183.03	105.83	-1.72	101.77
		CAU	-182.51	105.68	-1.72	101.77
		CAC	-183.55	108.55	-1.69	100.00
168	GGU	GAU	-179.67	109.75	-1.63	96.44
		GCU	-181.64	106.8	-1.7	100.59
		GUU	-179.25	96.07	-1.86	110.06
		AGU	-182.35	75.92	**-2.4**	142.01
		CGU	-179.56	112.23	-1.59	94.08
		UGU	-179.62	113.86	-1.57	92.89
170	CAA	CCA	-179.82	116.41	-1.54	91.12
		CGA	-183.48	108.33	-1.69	100.00
		CUA	-179.82	122.53	-1.46	86.39
		AAA	-179.07	131.06	-1.36	80.47
		GAA	-179.47	126.85	-1.41	83.43
		CAU	-179.88	123.01	-1.46	86.39
		CAC	-179.82	120.07	-1.49	88.16
172	ACU	AAU	-183.93	108.17	-1.7	100.59
		AGU	-184.7	107.92	-1.71	101.18
		AUU	-183.78	108.35	-1.69	100.00
		CCU	-182.74	110.2	-1.65	97.63
		GCU	-183.39	108.39	-1.69	100.00
		UCU	-183.66	107.83	-1.7	100.59
173	AAU	ACU	-183.86	108.42	-1.69	100.00
		AGU	-183.78	108.3	-1.69	100.00
		AUU	-183.85	108.24	-1.69	100.00
		CAU	-183.78	108.27	-1.69	100.00
		GAU	-183.78	108.34	-1.69	100.37
		UAU	-183.78	108.3	-1.69	100.00
		AAA	-183.06	108.56	-1.68	99.40
		AAG	-183.12	111.25	-1.64	97.04
174	GGU	GAU	-179.14	122.13	-1.46	86.39
		GCU	-179.18	121.55	-1.47	86.98
		GUU	-180.29	114.95	-1.56	92.30

		AGU	-180.83	97.38	-1.88	111.24
		CGU	-179.5	117.64	-1.52	89.94
		UGU	-180.61	113.73	-1.58	93.49
177	UAC	UCC	-181.97	96.55	-1.88	111.24
		UGC	-183.39	117.72	-1.55	91.71
		UUC	-183.62	76.28	**-2.4**	142.01
		AAC	-184.04	108.6	-1.69	100.00
		CAC	-183.02	109.52	-1.67	98.81
		GAC	-184.75	108.45	-1.7	100.59
All	Wildtype	None	-183.78	108.3	-1.69	100.00

*AMFE (Average minimum free energy in kcal/mol.) = Free energy/Ensemble diversity.

#Viral fitness (%) = (Mutant AMFE x 100) / Wild type AMFE (-1.69 kcal/mol.)

Table S2. *Viral fitness (%) for mutants based on the binding affinity calculations from the number of hydrogen bonds at the RBD-ACE2 receptor binding interface compared to the wild type (taken as 100%).

Substitution	K89	G118	Y121	N159	Q165	G168	Q170	T172	N173	G174	Y177
Ala	92.31	100.0	84.62	84.62	92.31	100.0	92.31	92.31	92.31	100.0	92.31
Cys	92.31	100.0	84.62	84.62	92.31	100.0	92.31	92.31	92.31	100.0	92.31
Asp	92.31	100.0	84.62	92.31	92.31	**107.7**	92.31	**115.4**	100.0	**115.4**	92.31
Glu	92.31	100.0	84.62	84.62	92.31	100.0	100.0	100.0	100.0	**107.7**	92.31
Phe	92.31	100.0	84.62	84.62	92.31	100.0	92.31	92.31	92.31	100.0	92.31
Gly	92.31	WT	84.62	84.62	92.31	WT	92.31	92.31	92.31	WT	92.31
His	92.31	100.0	84.62	92.31	92.31	100.0	92.31	92.31	92.31	**115.4**	92.31
Ile	92.31	100.0	84.62	84.62	92.31	100.0	92.31	92.31	92.31	100.0	92.31
Lys	WT	100.0	84.62	92.31	92.31	**107.7**	92.31	92.31	92.31	100.0	100.0
Leu	92.31	100.0	84.62	84.62	92.31	100.0	92.31	92.31	92.31	100.0	92.31
Met	92.31	100.0	84.62	84.62	92.31	100.0	92.31	92.31	92.31	100.0	92.31
Asn	92.31	100.0	84.62	WT	92.31	**115.4**	92.31	92.31	WT	**107.7**	92.31
Pro	92.31	100.0	84.62	84.62	92.31	100.0	92.31	92.31	92.31	100.0	92.31
Gln	92.31	100.0	84.62	92.31	WT	**107.7**	WT	92.31	92.31	**115.4**	92.31
Arg	92.31	100.0	84.62	92.31	92.31	**115.4**	92.31	92.31	92.31	**115.4**	**115.4**
Ser	92.31	100.0	84.62	84.62	92.31	**107.7**	92.31	100.0	92.31	**107.7**	100.0
Thr	92.31	100.0	84.62	84.62	92.31	**107.7**	92.31	WT	92.31	**107.7**	100.0
Val	92.31	100.0	84.62	92.31	92.31	100.0	92.31	92.31	92.31	100.0	92.31
Trp	92.31	100.0	84.62	84.62	92.31	100.0	92.31	92.31	92.31	100.0	92.31
Tyr	**107.7**	100.0	WT	84.62	92.31	**107.7**	92.31	92.31	100.0	**115.4**	WT

*Viral fitness (%) of mutants = (No. of interface H-bonds of mutant x 100) / No. of interface H-bonds of wild type.

NOTE: Amino acid substitutions with enhanced viral fitness are shown in bold font.

Table S3. Structural deviations that are >2.0 Å and <3.0 Å seen in mutants based on the C_{α} RMS deviations compared to the wild type at the site of mutation vs. off-sites. For deviations >3 please see Figure 6. For a complete histogram please see Figure 4.

Mutation	Lys89		Tyr121		Gly174	
	Mut. site	Off-site	Mut. site	Off-site	Mut. site	Off-site
Ala	<2.0 Å	<2.0 Å	0.9 Å	2.1Å (149), 2.2Å (192) & 2.3Å (193)	<2.0 Å	<2.0 Å
Cys	<2.0 Å	<2.0 Å	0.9 Å	2.1Å (149), 2.2Å (192) & 2.3Å (193)	<2.0 Å	<2.0 Å
Asp	<2.0 Å	<2.0 Å	0.9 Å	2.1Å (149), 2.2Å (192) & 2.3Å (193)	<2.0 Å	<2.0 Å
Glu	<2.0 Å	<2.0 Å	0.9 Å	2.1Å (149), 2.2Å (192) & 2.3Å (193)	<2.0 Å	<2.0 Å
Phe	<2.0 Å	<2.0 Å	0.9 Å	2.1Å (149), 2.2Å (192) & 2.3Å (193)	<2.0 Å	<2.0 Å
Gly	<2.0 Å	<2.0 Å	0.9 Å	2.1Å (149), 2.2Å (192) & 2.3Å (193)	<2.0 Å	<2.0 Å
His	<2.0 Å	<2.0 Å	0.9 Å	2.1Å (149), 2.2Å (192) & 2.3Å (193)	<2.0 Å	<2.0 Å
Ile	<2.0 Å	<2.0 Å	0.9 Å	2.1Å (149), 2.2Å (192) & 2.3Å (193)	<2.0 Å	<2.0 Å
Lys	<2.0 Å	<2.0 Å	0.9 Å	2.1Å (149), 2.2Å (192) & 2.3Å (193)	<2.0 Å	<2.0 Å
Leu	<2.0 Å	<2.0 Å	0.9 Å	2.1Å (149), 2.2Å (192) & 2.3Å (193)	<2.0 Å	<2.0 Å
Met	0.5 Å	2.1Å (150)	0.9 Å	2.1Å (149), 2.2Å (192) &	<2.0 Å	<2.0 Å

SUPPLEMENTARY DATA

				2.3Å (193)		
Asn	0.5 Å	2.1Å (150)	0.9 Å	2.1Å (149), 2.2Å (192) & 2.3Å (193)	<2.0 Å	<2.0 Å
Pro	0.5 Å	2.1Å (150)	0.9 Å	2.1Å (149), 2.2Å (192) & 2.3Å (193)	<2.0 Å	<2.0 Å
Gln	0.5 Å	2.1Å(150)	0.9 Å	2.1Å (149), 2.2Å (192) & 2.3Å (193)	<2.0 Å	<2.0 Å
Arg	0.5 Å	2.1Å (150)	0.9 Å	2.1Å (149), 2.2Å (192) & 2.3Å (193)	<2.0 Å	<2.0 Å
Ser	0.2 Å	2.6Å (38) & 2.9Å (53)	0.9 Å	2.1Å (149), 2.2Å (192) & 2.3Å (193)	<2.0 Å	<2.0 Å
Thr	0.2 Å	2.6Å (38) & 2.9Å (53)	0.9 Å	2.1Å (149), 2.2Å (192) & 2.3Å (193)	<2.0 Å	<2.0 Å
Val	0.5 Å	2.1Å (150)	0.9 Å	2.1Å (149), 2.2Å (192) & 2.3Å (193)	<2.0 Å	<2.0 Å
Trp	0.5 Å	2.1Å (150)	0.9 Å	2.1Å (149), 2.2Å (192) & 2.3Å (193)	1.29 Å	2.3Å (5), 2.12Å (56), 2.3Å (192) & 2.2Å (193)
Tyr	0.5 Å	2.1Å (150)	0.9 Å	2.1Å (149), 2.2Å (192) & 2.3Å (193)	<2.0 Å	<2.0 Å

NOTE: Values in parentheses indicate the position of the deviation.
